# Assessment of avian health status: suitability and constraints of the Zoetis VetScan VS2 blood analyser for ecological and evolutionary studies

**DOI:** 10.1242/bio.060009

**Published:** 2023-07-24

**Authors:** Ye Xiong, Michael Tobler, Arne Hegemann, Dennis L. Hasselquist

**Affiliations:** Department of Biology, Lund University, Ecology Building, SE-223 62 Lund, Sweden

**Keywords:** Blood physiology, Acute stress, Measurement quality, Repeatability, Eco-physiology, Veterinary sciences

## Abstract

Biochemical analyses of blood can decipher physiological conditions of living animals and unravel mechanistic underpinnings of life-history strategies and trade-offs. Yet, researchers in ecology and evolution often face constraints in which methods to apply, not least due to blood volume restrictions or field settings. Here, we test the suitability of a portable biochemical analyser (Zoetis VetScan VS2) for ecological and evolutionary studies that may help solve those problems. Using as little as 80 µl of whole-bird blood from free-living Jackdaws (*Corvus monedula*) and captive Zebra Finches (*Taeniopygia guttata*), we show that eight (out of 10) blood analytes show high repeatability after short-term storage (approximately 2 h) and six after 12 h storage time. Handling stress had a clear impact on all except two analytes by 16 min after catching. Finally, six analytes showed consistency within individuals over a period of 30 days, and three even showed individual consistency over a year. Taken together, we conclude that the VetScan VS2 captures biologically relevant variation in blood analytes using just 80 µl of whole blood and, thus, provides valuable physiological measurements of (small) birds sampled in semi-field and field conditions.

## Teaser

**Summary:** Measuring multiple parameters of physiology in wild animals remains difficult. We evaluate a veterinary device the VetScan blood analyser, including effects of storage time and handling stress, typical during (semi-)field conditions.

## INTRODUCTION

Measuring physiology in ecological and evolutionary studies has become increasingly popular over the last few decades, partly because the possibilities for measuring physiological parameters in (small) free-living animals have advanced substantially. Several tools and techniques have become available to measure a multitude of physiological parameters related to metabolism, nutritional status, hormones and immune function, for example, and these tools can potentially also be useful in unravelling physiological mechanisms underlying behaviour and life-history strategies. Understanding how physiology shapes, or is shaped by, for example, an organism's reproductive or migratory decisions is crucial for our understanding of how different strategies can evolve (Andreasson et al., 2020; Evans and Gustafsson, 2017; Hasselquist et al., 2007; Hegemann et al., 2019; McWilliams et al., 2021). Moreover, studying physiological variation in response to biotic and abiotic stressors can help us to understand and predict how animals can cope with stressful conditions including potential effects of global change (Enders et al., 2015; Lettoof et al., 2021).

There is a wide range of assays that measure different aspects of physiology, including hormones (Fletcher et al., 2018; Huber et al., 2017; Wingfield and Farner, 1975), oxidative stress (Costantini, 2014; Gutiérrez et al., 2019; Vágási et al., 2019), immune function (Adamo, 2004; Demas et al., 2011; Hasselquist et al., 2001; Matson et al., 2005) and markers of ageing such as telomere length (Chaney et al., 2003; Criscuolo et al., 2009). Most of these assays require a blood sample. Because the blood volume that can be sampled from small species is often limited, this can restrict the number of assays that can be performed, and hence, the number of physiological parameters that can be measured simultaneously. Many studies therefore often focus on only one physiological system.

Measures of specific aspects of animal physiology can provide estimates of individual quality and health, and the effectiveness of the body to react to environmental stimuli. Compared to assays that target specific physiological systems, simultaneous measurement of multiple biochemical blood analytes could give an overall and hence more comprehensive picture of an animal's health status. However, even though simultaneous measurement of multiple blood analytes is widespread in veterinary science (Adams et al., 2022; Ammersbach et al., 2015; Christopher and O'Neill, 2000; Ruiz-Jimenez et al., 2021) and has been used in dietary studies (e.g. Basile et al., 2020; 2021), there are still relatively few studies applying such methods in an eco-physiological context, especially in wild populations (but see Davies et al., 2013; Greenacre et al., 2008; Li et al., 2019; Schoepf et al., 2017). One likely reason for this is that such analyses hitherto often required a range of different assays, large blood volumes or immediate analysis in the laboratory (Heins et al., 1995; Kachhawa et al., 2017; Männistö et al., 2007). Furthermore, analyses with conventional biochemistry analysers (e.g. Cobas analyser; Ammersbach et al., 2015; Ruiz-Jimenez et al., 2021) or metabolomics studies (which allow simultaneous analyses of a very large number of chemical analytes; e.g. Basile et al., 2020; 2021) are less feasible for researchers in the fields of ecology and evolution, as they require expensive equipment, trained personnel and often also that sampling is done in close proximity to the laboratory. More recently, portable biochemical blood analysers that can measure a range of analytes simultaneously have been used in ecological studies (Atkins et al., 2010; Awerman and Romero, 2010; Peruffo et al. 2016; Ruiz-Jimenez et al., 2021; Schoepf et al., 2017). These instruments can be set up in temporary laboratories close to field sites and, thus, be used in field settings. As these analysers are relatively inexpensive, do not demand a high hygiene working environment or specialized personnel for running the assays, they may be a valuable alternative for conventional laboratory analyses. However, such methods are still relatively unknown to most ecologists and evolutionary biologists and there is a lack of knowledge regarding their practical applicability in field settings that pose different challenges from studies on caged and domesticated animals.

Here, we test the suitability of a portable biochemical blood analyser, the VetScan VS2 (Zoetis), to evaluate its usefulness in eco-physiological studies using avian blood. The VetScan is designed for quick analysis of multiple health-related blood parameters in veterinary applications on different animal groups (mammals, birds and reptiles). The Vetscan has been used previously in several studies on chronic stress and reproductive costs (Awerman and Romero, 2010; Lettoof et al., 2021; Schoepf et al., 2017; Sørensen et al., 2021) and comparisons of the Vetscan analyser with reference laboratory analysers for biochemistry show good agreement for the majority of parameters in different bird species (Adams et al., 2022; Ammersbach et al., 2015; Ruiz-Jimenez et al., 2021). However, to the best of our knowledge, no study has tested the consistency over time of the parameters measured by the VetScan analyser, nor studied the sensitivity of different parameters to handling stress, time of day for sampling and sample storage time. This type of knowledge is essential, however, for researchers when deciding whether to invest in a portable blood analyser. Hence, the aim of our study was to test the suitability of the VetScan VS2 blood analyser under settings typical for researchers conducting ecological and evolutionary studies, and to evaluate whether there are constraints related to situations that evolutionary ecologists experience. Specifically, we wanted to (1) test whether Vetscan measurements are repeatable between rotors under conditions typical for ecological studies, (2) assess the effects of storage time and time of day on measurement precision, (3) investigate to what extent handling stress may confound analyte values, and (4) measure individual consistency of measurements over longer time periods (weeks, months).

## RESULTS

### Measurement repeatability

Analyses of the same blood sample within 15 min showed high repeatability for all blood parameters (R=0.84-0.99) except sodium (Na+) (Table 1). Repeatability of Na+ was significant, but the R-value was only 0.52 and therefore considerably lower than that of the other parameters. Given this, we concluded that sodium measurements are not reliable enough and we did not use them in any further analyses.

**Table 1.**
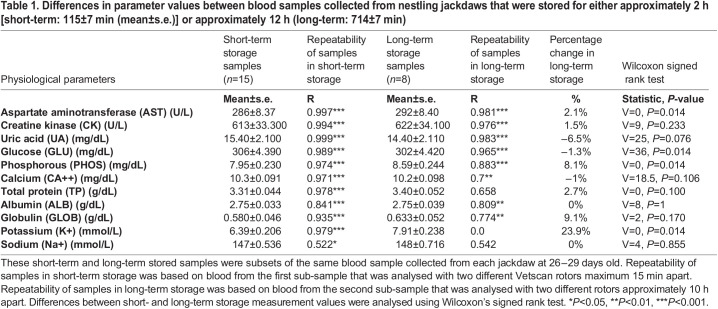
Differences in parameter values between blood samples collected from nestling jackdaws that were stored for either approximately 2 h [short-term: 115±7 min (mean±s.e.)] or approximately 12 h (long-term: 714±7 min)

### Effects of storage time and time of day

For seven of the remaining 10 blood analytes (excluding BA and Na+) that could be properly analysed, there was no association between short-term storage and measurement values in nestling jackdaws [all *F*<1.48, degrees of freedom (d.f.)=13, *P*>0.25; Table 2]. However, values of aspartate aminotransfrase (AST) and ALB were negatively affected by short-term storage [AST: β=-0.625, s.e.=0.272, *P*=0.04; ALB: β=-0.003, standard error (s.e.)=0.001, *P*=0.01]. For potassium (K+) there was a trend for a similar association (β=-0.013, s.e.=0.007, *P*=0.07).

**Table 2.**
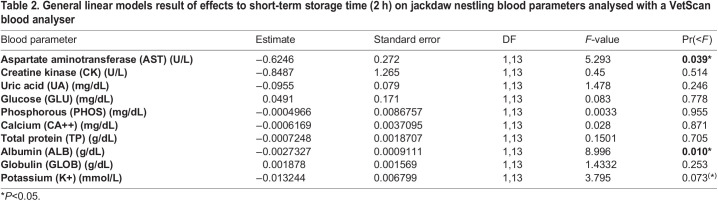
General linear models result of effects to short-term storage time (2 h) on jackdaw nestling blood parameters analysed with a VetScan blood analyser

**Fig. 1.**
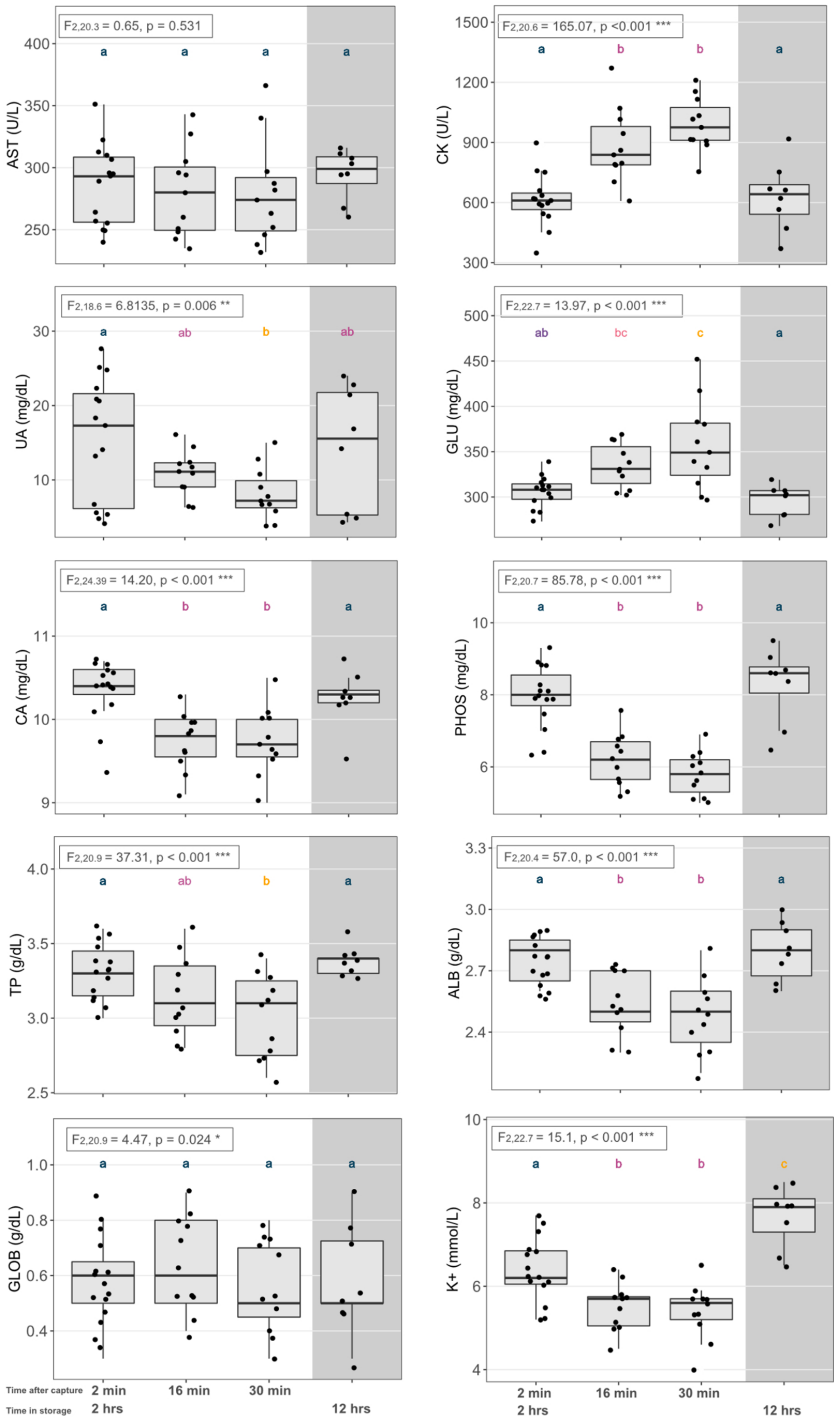
**VetScan measurement results from handling stress and the 12 h storage experiment conducted on jackdaw *Corvus monedula* nestlings.** The x-axis labels denote the time from capture to when the blood sample was taken [2 min (baseline), *n*=15; 16 min, *n*=11; and 30 min (stress samples), *n*=11] and the maximum storage time for jackdaw samples (12 h, shaded area). See Materials and Methods for detailed handling and storage protocols. The y-axis denotes the range of the values for the different blood analytes. The box in the upper left corner of each panel reports the statistics of the repeated measures analyses for the 2-min, 16-min and 3-min samples. Different letters above the error bars reflect significant differences (*P*<0.05) and are based on Tukey post-hoc tests for the repeated measures. Statistical differences between the measurement values analysed after 2 h and after 12 h, respectively, were based on Wilcoxon signed rank tests. The full form of blood parameters: aspartate aminotransferase (AST), creatine kinase (CK), uric acid (UA), glucose (GLU), phosphorous (PHOS), calcium (CA^++^), total protein (TP), albumin (ALB), globulin (GLOB), and potassium (K^+^).

Long-term storage (for approximately 12 h at 4°C) did not affect four of the blood parameters: the concentrations of ALB, creatine kinase (CK), calcium (CA), and globulin (GLOB) were stable over time. However, long-term storage had a significant effect on the other six blood analytes. Measurement values of the same nestling jackdaw blood sample decreased for UA and glucose (GLU), but increased for AST, K+, phosphorus (PHOS), and TP (Table 1, Fig. 1). Notably, the change for AST and K+ values is in the opposite direction compared to the short-term storage effect. A repeatability analysis for the same samples measured after 2 h and 12 h, respectively, showed that eight out of 10 blood analytes were highly repeatable between measurements even after 12 h (Table 1). This means that the relative change in measurement values over time for AST, UA, GLU and PHOS was of the same direction and magnitude in the different samples. Only TP and K+ values showed low and non-significant repeatability.

UA values of blood samples collected from jackdaw nestlings in the morning were higher compared to values of samples collected in the afternoon (β=−0.001, s.e.=0.0003, *P*=0.020). For the other nine blood analytes, there was no significant difference between samples collected in the morning and in the afternoon (all *F*<1.48, d.f.=13, *P*>0.25).

### Effects of handling stress

Handling stress influenced eight out of the 10 blood analytes we could investigate. The exceptions were AST and GLOB which did not change over the repeated sampling period of 30 min (see Fig. 1 for statistical results). Of the eight blood analytes that were affected by handling stress, two (CK, GLU) showed significant increases in concentration over the time period when birds were exposed to handling stress, whereas six (UA, CA, PHOS, TP, ALB, K+) showed significant decreases in concentration (Fig. 1). The blood analytes which showed the strongest changes over the 30 min stress period were CK, UA, PHOS and GLU with +66%, −46%, −28% and +16% change, respectively (Fig. 1). Interestingly, the effect of handling stress for GLU (increase) and PHOS (decrease) was opposite to the 12 h storage effect (Fig. 1).

### Individual consistency of measurements over longer time periods

**Fig. 2.**
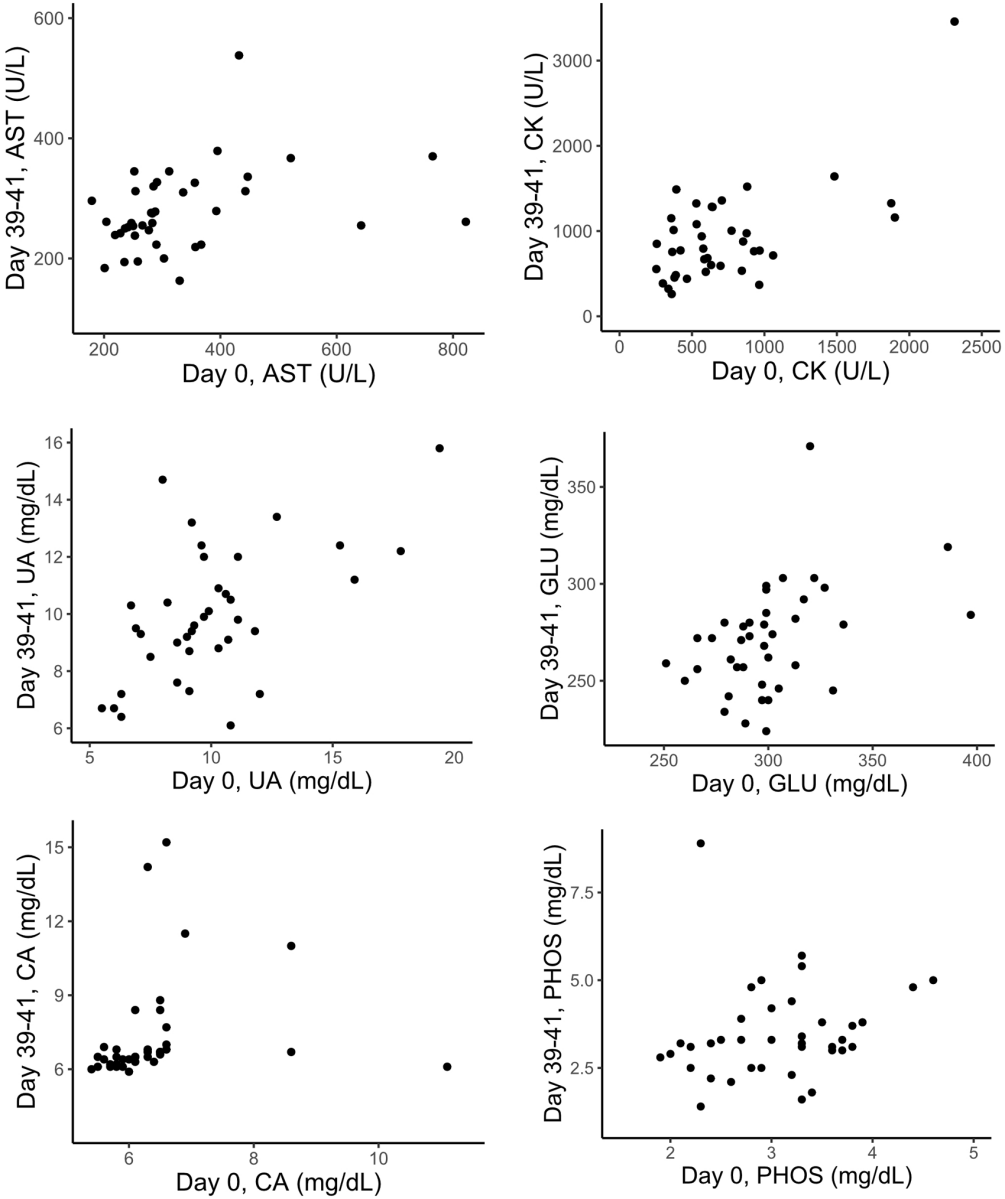
**Relationship between physiological values from a VetScan blood analyser when comparing the initial sample (day 0) and the values of the samples collected approximately 40 days later from the same adult zebra finches (CK and CA, *n*=37; AST, UA, GLU and PHOS, *n*=38) that were kept in groups in outdoor enclosures.** For statistical analyses, see Table 3.

**Fig. 3.**
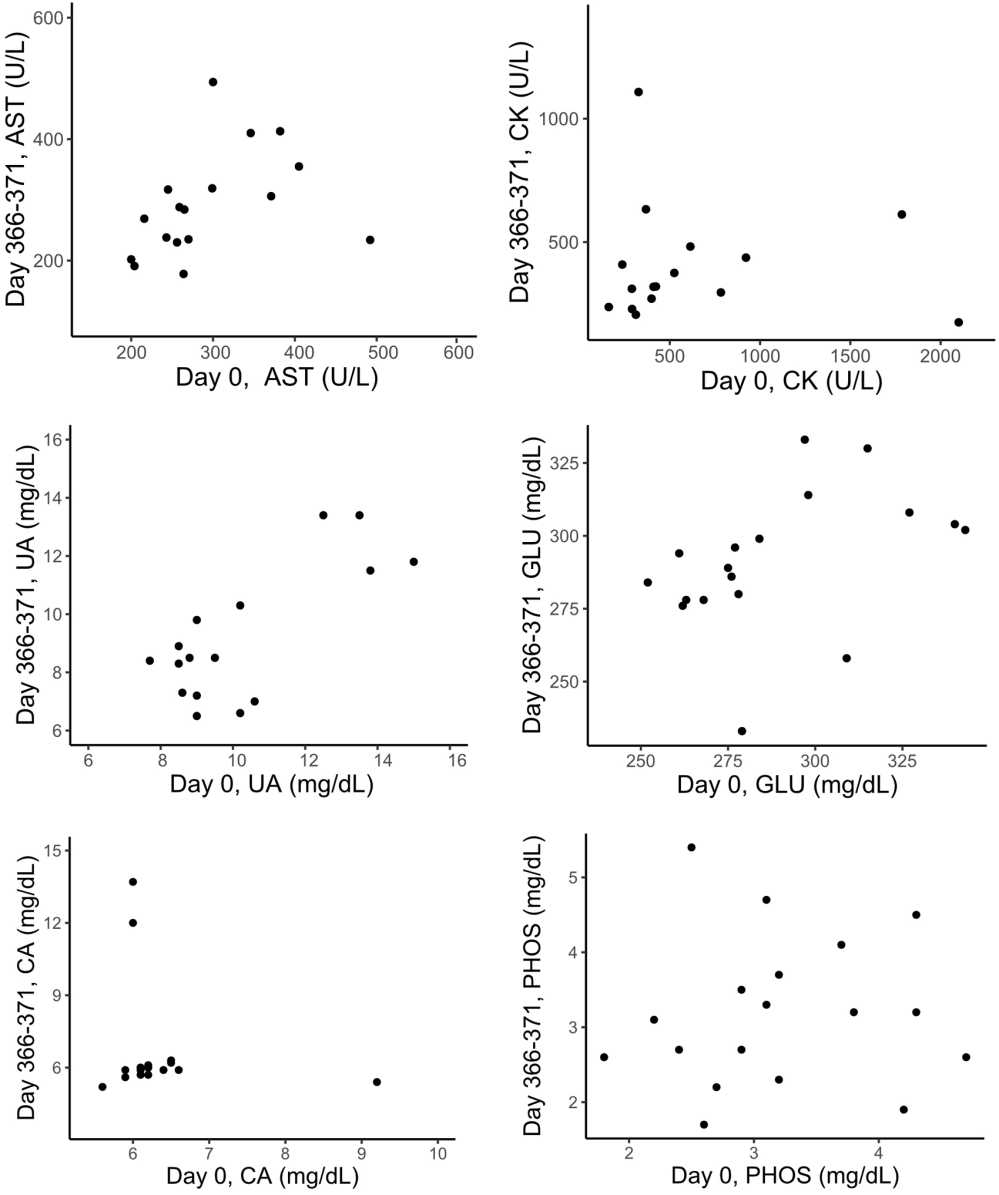
**Relationships between the values for physiological parameters as analysed by a VetScan blood analyser and comparing the initial sample (day 0) and the values of the samples collected approximately 1 year later from the same adult zebra finches (CK, *n*=16; AST, UA, GLU, CA and PHOS, *n*=17) that were kept in groups in outdoor enclosures.** For the statistical analyses, see Table 3.

Five out of seven blood analytes in zebra finches showed significant positive relationships between the initial parameter value (day 0) and the parameter value measured approximately 40 days later (Table 3, Fig. 2). When we tested for similar relationships between the initial values and the values obtained approximately 1 year later, three of the blood analytes (AST, UA, GLU) still showed a significant positive relationship (Table 3, Fig. 3). Repeatabilities for the same blood parameters are also listed in Table 3. Measures of AST, CK and UA showed significant repeatabilities over the 40-day-period, and UA and GLU showed significant repeatabilities for measurements that were 1 year apart.

**Table 3.**
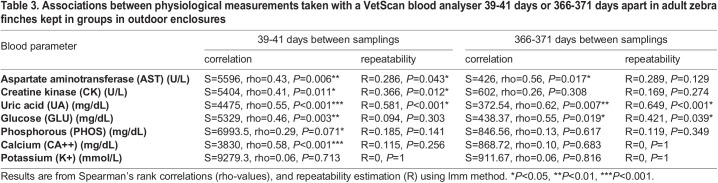
Associations between physiological measurements taken with a VetScan blood analyser 39-41 days or 366-371 days apart in adult zebra finches kept in groups in outdoor enclosures

## DISCUSSION

Understanding patterns among species or individuals in ecological and evolutionary studies often benefits from including physiological parameters. A limitation of conventional laboratory analyses is that they are often not accessible for researchers collecting samples on wild animals due to both financial and logistical limitations. Here, we evaluated the suitability of a portable blood analyser when used in a field setting.

### Measurement stability and storage effects

We found that BA fell below the detection limit of the machine and therefore cannot be used in our study species, and similar results have been obtained in other studies (e.g. Ammersbach et al., 2015; Ruiz-Jimenez et al., 2021). Moreover, measurement values of Na+ only showed moderate repeatability even for analyses that were based on the same jackdaw blood sample and performed within 13 min of each other. This is congruent with other studies showing poor agreement in Na+ measures between the VetScan analyser and conventional biochemistry analysers (Adams et al., 2022; Ammersbach et al., 2015; Ruiz-Jimenez et al., 2021). Hence, we suggest caution when analysing sodium values with the VetScan in studies on passerines. The remaining 10 blood parameters showed high repeatability and, thus, high between-rotor stability showing that rotor differences contribute very little to the variation in blood measurements.

Both short-term (2 h) and long-term (12 h) storage time affected some of the blood analytes. Previous studies have looked at the effect of time in blood measurements taken with the VetScan after 20 and 40 min and found little changes in the values (Ruiz-Jimenez et al., 2021; Adams et al., 2022). Interestingly, AST and K+ showed a decrease in concentration over the first 2 h after sampling, but an increase in concentration over 12 h. We do not have an explanation for this somewhat puzzling result, although a possibility is that the subsequent increase could be associated with the suggested increase in hemolysis in samples that were stored for 12 h. Increased degradation of red blood cells possibly could free up more intracellular AST and K+ resulting in higher values measured by the rotor (Azman et al., 2019; Heireman et al., 2017; Hoppes et al., 2015). Our results agree with other studies that have measured effects of sample storage time on measurements of blood parameters, such as PHOS (increase), GLU and UA (decrease) (Christopher and O'Neill, 2000; Hoppes et al., 2015; Lee et al., 2016; Turchiano et al., 2013). The increase in PHOS levels in plasma or serum samples have been considered as an indicator of delayed handling after blood collection, because the prolonged contact of plasma and serum increases the risk for cell hemolysis and leakage of intracellular constituents (Lee et al., 2016; Oddoze et al., 2012). The significant decline of GLU is likely due to glycolysis. In unprocessed human blood, GLU decreases by 5–7% per hour (Turchiano et al., 2013), and a higher decline in cat (*Felis silvestris catus*) serum than plasma samples has been found (Christopher and O'Neill, 2000). Our results agree with a previous study on psittacine birds that found a decline in GLU levels from 3 h after blood sampling (Hoppes et al., 2015). For UA, the same study (Hoppes et al., 2015) found no change in UA concentration 6 h after blood collection, but a significant decrease after 24 h, which is congruent with our results showing a decrease after 12 h. Thus, different analytic assays seem to detect similar effects on blood analyte measurements caused by prolonged sample storage time. However, for one parameter, TP, Hoppes et al. (2015) found no changes in measurement values when stored at ambient temperature (25–26°C) for up to 24 h. This might suggest that for analyses of TP, the Vetscan machine is more sensitive to storage time.

Given the sensitivity of these parameters to prolonged storage time, samples should ideally be analysed within 2 h of sampling so that absolute values can be compared across studies. However, irrespective of the change in absolute levels, it is clear from our results that eight of the 10 measured parameters still showed high repeatability (R=0.70-0.98) between time points even when measured 12 h apart. This means that the relative change was the same in each sample. Hence, as long as the samples are treated equally, i.e. analysed in the same order and with rather similar time delays (note that it takes 13 min to run one samples, thus for 40 samples it would take about 9 h to complete the lab work), it is still possible to compare samples that have been collected over a whole day, with the first samples being stored for up to 12 h.

### Effects of handling stress

Various studies have shown that blood GLU levels increase, whereas UA levels (a product of protein catabolism) decrease in response to acute stress (Davies et al., 2013; Deviche et al., 2016; Li et al., 2019). Moreover, a range of other physiological parameters, such as fatty acids (Viblanc et al., 2018), antioxidants (Cohen et al., 2007) and immune parameters (Buehler et al., 2008; Matson et al., 2006; Nordfjäll et al., 2005; Zylberberg, 2015) have also been shown to change in response to stress.

Our results show that eight out of 10 parameters that could be reliably measured with the VetScan were significantly affected by handling stress, and that the response was rapid, already apparent after 16 min. Thus, it is possible to assess rapid changes in blood chemistry with the VetScan. However, it also highlights the fact that handling stress, which occurs during capture and sample collection, has the potential to affect the test results of most analytes that can be measured with the VetScan. If possible, blood samples should therefore be collected as quickly as possible after capture (ideally within 3 min), and prolonged handling should be avoided.

The stress-related decrease in UA, and the increase in GLU and CK, are consistent with changes in these parameters reported in other studies on birds that were exposed to short-term stress (Deviche et al., 2016; Li et al., 2019; Scope et al., 2002). We found a 46% decrease in UA caused by 30 min handling stress in nestling jackdaws, and this result is consistent with other studies on tree sparrows (*Passer montanus*) adults (Li et al., 2019) and adult male rufous-winged sparrows (*Peucaea carpalis*) (Deviche et al., 2016). Both CK (+66%) and GLU (+16%) showed a rapid response with significant increases from 3 to 30 min after capture in our study. A similar study in racing pigeons (*Columba livia domestica*) found increases of 90% in CK and 10% in GLU, a decrease of –35% in UA but no changes in AST and TP after 3 h kept in transport cages (Scope et al., 2002). UA is a non-enzymatic antioxidant used as an indicator for renal health, and increasing tissue uptake of UA during capture stress may be beneficial to improve antioxidant capacity (Cohen et al., 2007; Deviche et al., 2014; Sautin and Johnson, 2008; Strazzullo and Puig, 2007). This further validates the usefulness of the VetScan to measure physiological responses to short-term stress.

### Individual consistency of blood analytes

It is well known that behavioural and life-history strategies are associated with physiological status (Flatt and Heyland, 2011). Investigating the intra-individual consistency of physiological traits can be informative because repeatability of a trait is usually assumed to set the upper limits to heritability in the broad sense (i.e. including both genetic and environmental variation of the trait) (Dohm, 2002; Falconer and Mackay, 1996). Significant repeatability of a trait suggests that trait expression is a characteristic of the individual and that there may be some genetic variation underlying the trait. Hence, it can allow inferences about how effective natural selection can be on altering trait expression over time (Dohm, 2002). Blood analytes that show individual consistency, especially over longer time periods, may suggest that individual levels of these analytes can have some heritable component and, thus, may be a target of selection. Physiological traits that are significantly repeatable over time may also represent a mechanistic constraint for adaptive behavioural strategies or reproductive responses and therefore likely also survival. Our measures on zebra finches show that some of the blood analytes (CK, CA, UA, GLU, and AST; Table 3) were relatively consistent over time, and the latter three even over a period of 1 year (although note that UA is sensitive to prolonged handling stress and GLU affected by time of day of sampling). Hence, we argue that these VetScan measures may be useful to track how individual differences in physiological/general health status are maintained over time, how they relate to individual differences in reproductive output or senescence, and possibly also to characterise physiological profiles that can be associated with animal personalities.

### Whole blood versus plasma

In this study, we focus on analyses of whole blood only. It is possible to run plasma samples on the VetScan rotors (Atkins et al., 2010; Lettoof et al., 2021), but the minimum amount of fluid required for successful analysis is still 80 µl. This means that plasma analyses are not feasible for small organisms from which less than 200 µl of blood can be collected. However, studies on chickens and parrots show that measurements from whole blood and/or blood saline mixture are highly comparable to measurements from plasma samples (Greenacre et al., 2008; Morales et al., 2020; Ruiz-Jimenez et al., 2021). Hence, results from our study related to handling stress or sample storage time should also be valid for plasma samples and opens the possibility to use the VetScan analyser for already collected plasma samples that have been stored or in situations where direct analyses are not feasible.

Our results show that VetScan VS2 gave high repeatability in measurements using as little as 80 µL of fresh whole blood or 1/5 mixture of saline and whole blood. Hence, the device can be helpful in getting a more complete picture of an animal's health status even in small birds. Depending on the purpose of study, VetScan can provide relatively stable measurements of most of the physiological parameters it has been designed to measure. However, there are also certain limitations that need to be considered when deciding on study design, e.g. related to handlings stress and blood sample storage time. In addition, some of these limitations are also highly relevant for veterinary studies and sampling protocols, e.g. for establishing reference intervals for blood parameters, should aim to control for these. Still, we suggest that the VetScan blood analyser can be a useful additional tool for eco-physiological (field) studies.

## MATERIALS AND METHODS

### Study species

In our study, we used two bird species: free-living nestling jackdaws (*Corvus monedula*) and captive adult zebra finches (*Taeniopygia guttata*).

Samples of jackdaw nestlings were collected in June 2020 from a wild nest box breeding colony at Revingehed (N 55° 43′ 1.938″ E 13° 26′ 22.477″) in southern Sweden (Aastrup and Hegemann, 2021; Aastrup et al., 2023). The relatively large size of jackdaw nestlings (223.1±17.9 g when blood samples were taken at an age of 26 to 29 days) allowed collection of a larger blood volume and repeated sampling over a short period of time. This enabled us to test the performance of the VetScan VS2 with respect to repeatability between VetScan rotors under natural settings, the effects of shorter (2 h at 4°C) versus longer (12 h at ca 4°C) sample storage time, and whether handling stress affects the parameter values.

To assess within-individual consistency of VetScan parameters over longer periods of time, we sampled captive zebra finches housed at the field station at Stensoffa run by Lund University three times: at the beginning of the study, after 6 weeks and after 12 months. Zebra finch adults moved in from outdoor aviary 1 day before the blood sampling started, birds were housed indoors with two same-sexed birds per cage (59×33×57 cm) at constant temperature (22±2°C) and photoperiod (12 L: 12D) for 6 weeks. Thereafter they were housed in flocks of approximately 60 birds in sex-specific groups in outdoor aviaries (3.5×3.7×2.3 m). A year after the first measurement, i.e. at the time of the third measurement, birds were again housed indoors under the same conditions as during the first sampling period and moved indoors 3 days before the start of blood sampling. Birds had *ad libitum* access to a mixed seed diet (Franks Zoofor AB), drinking water, and sepia shells (for calcium), at all times.

### VetScan instrument and physiological health

We used analysis rotors for the VetScan that have been optimized for bird and reptilian blood (Avian/Reptilian Profile Plus). One avian/reptile VetScan rotor can examine up to 12 different blood analytes (more details see, https://www2.zoetisus.com/content/_assets/docs/Diagnostics/package-inserts/VETSCAN-Avian-Reptilian-Profile-Plus-Package-Insert-LBL-02420.pdf) simultaneously on the same blood sample and may therefore give a quick composite measure of a bird's physiological health status. It requires relatively small amounts of whole blood (less than 120 µl) that can be taken from most bird species, including relatively small songbirds. According to the manufacturer's description, results from a VetScan analysis can offer an evaluation of electrolyte status, nutritional level, fluid balance, liver integrity, and kidney and renal function. Thus, it provides both an assessment of specific organ functions, as well as an overall health assessment (see pdf file above for a list of the health functions reflected by the blood parameters).

We were not aiming for a technical validation of the reliability of the chemical analyses per se, as this type of validation has been done in other studies (see above). Moreover, we were not interested in comparing the VetScan VS2 machine to other portable blood analysers. Our sampling procedures were instead designed to test the suitability of the VetScan analyser for studies conducted in field settings and to identify potential limitations with regards to sampling protocols.

### Sampling procedure

For the jackdaw blood-sampling, we randomly chose one nestling from 15 nest boxes in the colony on 2-3 June 2020. Blood was collected from the jugular vein with a sterile discardable syringe and transferred into an Eppendorf tube coated with lithium heparin. Lithium heparin was used instead of the more commonly used sodium heparin because sodium heparin interferes with some of the measurements in the Vetscan analyses (https://www2.zoetisus.com/content/_assets/docs/Diagnostics/package-inserts/VETSCAN-Avian-Reptilian-Profile-Plus-Package-Insert-LBL-02420.pdf). At blood sampling, we also measured nestling body mass (to the nearest 0.1 g). We collected a first sample of 300–400 µl from all 15 individuals within 3 min (136±27 s) after removal from the nest box. Subsequently, we obtained another 100 µl from 12 individuals 16 min (16±1 min) after the first sampling, and a third sample of 100 µl from 14 individuals 30 min (30±1 min) after the first sampling (in total, 11 individuals were sampled twice each, and 11 individuals were sampled three times each). Jackdaw blood samples were taken in the morning (between 09:29 and 10:41 h, *n*=7 nestlings) or in the afternoon (between 13:13 and 14:04 h, *n*=8 nestlings). All blood samples were stored immediately on ice in the field and then transported to the field station Stensoffa (3.6 km away) where they were stored at 4°C in a fridge until further analysis with the VetScan.

Zebra finches were sampled between 08:50 and 12:00 h over 2–4 consecutive days on three occasions (5–8 Mar 2019, 15–16 Apr 2019 and 9–11 Mar 2020). Blood (75 µl) was collected from the jugular vein with a sterile discardable syringe and transferred into an Eppendorf tube coated with lithium heparin (see above). In total, we collected 114 samples of 57 zebra finches (39 individuals sampled twice 39–41 days apart, and 18 additional individuals sampled two times 366–371 days apart). The zebra finch study included 29 males and 28 females that were between 8–77 months old at first sampling (23.8±1.4 months). Blood samples were immediately stored in a fridge at 4°C until further use on the same day. All zebra finch samples were also directly analysed at the field station.

### Measurement procedure

The recommendation by the manufacturer is to use a sample volume of 100 µl, but preliminary testing showed that the instrument also performs well with 80 µl (but not with smaller volumes). Hence, in this study, we used for each analysis and rotor 80 µl of fresh blood from jackdaws. For zebra finches our ethical permit did not allow us to collect more than 75 µl blood per sampling. Hence, we used the same approach as Schoepf et al. (2017) diluting the blood with saline solution. We diluted 75 µl of zebra finch blood with 15 µl 0.9% saline solution by pipette, and then we used 80 µl of this blood-saline mixture in the Vetscan analysis. Putting less than 80 µl blood/blood mixture into a Vetscan rotor will cause measurement failure with the instrument not completing the measurements. Thus, a minimum volume of 80 µl is essential for instrument function.

The VetScan sampling procedure is as follows: 80 µl of the sample (whole blood for jackdaws or blood/saline-mixture for zebra finches) was pipetted into an Avian/Reptilian Profile Plus rotor at an angle of about 45°, which facilitates even distribution of the sample in the rotor's blood collection compartment. The rotor was then immediately placed on the VetScan tray for spinning and analysis. Spinning results in separation of blood cells and plasma, thereafter a standardized quantity of plasma is mixed with diluent (contained by the rotor). The mixture then flows into the reaction cuvettes along the rotor perimeter where the chemical reactions take place and are measured photometrically. All samples were run on the same VetScan instrument, and as analysis of one sample takes 13 min, all blood samples were processed within 13 h. For jackdaws, measurement values for bile acids (BA) were below the detection threshold, and we therefore only report results for the 11 remaining blood parameters. For zebra finches, only seven (of 12) blood analytes were both above the detection threshold and passed the internal quality check of the machine. Internal quality checks (for hemolysis, lipemia, and icterus) are performed by the VetScan machine to ensure accurate results, and when the analyser detects a problem, it will either suppress certain chemistry results or cancel the run.

In this study, we restricted the analyses to whole blood samples even though (frozen) plasma samples can also be run on the VetScan (Atkins et al., 2010; Lettoof et al., 2021). However, obtaining sufficient plasma for small birds such as zebra finches is not feasible unless they are sacrificed, which is usually not desirable in ecological studies.

### Measurement repeatability

To study the repeatability (reproducibility) of the Vetscan measurements for each analyte, we used the large (300–400 µl) blood samples collected from jackdaw nestlings within 3 min after removal from the nest box and run blood from the same sample on two different rotors immediately after each other (average: 13.4±0.3 min, range 12–15 min between starting the two analyses). This allowed us to assess whether there is any significant variation due to differences in rotor quality or instrument instability when the machine is run under non-standard laboratory conditions. Most previous studies have routinely run one sample per individual and the manufacturer claims high repeatability between rotors. Only two studies have run blood from the same sample repeatedly with the same blood analyser (Ammersbach et al., 2015; Ruiz-Jimenez et al., 2021). However, these studies have not assessed repeatability of the measurements, i.e. how much of the total variance in measurements is due to individual variation rather than due to variation in rotor or instrument performance. To our knowledge, this has not been tested previously. We therefore conducted such a test in this study, as we think that knowledge about rotor stability in non-standard lab environments is critical and adds to the studies comparing different analysers.

### Effects of storage time

In many studies on wild animals, it is difficult to bring blood samples to the lab immediately and instead samples need to be stored on ice in the field before returning to the lab. To test whether storage time influences the parameter values of blood analytes, we analysed jackdaw samples that were kept cold over varying periods of time. We ran two different analyses on the jackdaw samples. First, we analysed whether ‘short-term’ storage time (time between the first blood sampling and the first blood analysis with the VetScan VS2; mean: 114.7 min, range: 71–161 min) predicted parameter values. Second, we evaluated whether parameter values of the same sample differed if they were stored short-term or long-term (in the latter case, the samples were stored in the fridge for approximately 12 h; mean: 713.8 min, range: 687–745 min). Keeping the blood samples at 4°C in the fridge for 12 h mimics a common situation where samples need to be kept on ice in the field for a large part of the day before being transported to a laboratory for analyses.

### Effects of time of day of sampling

To assess whether time of day of sampling affected parameter values measured with the VetScan, we compared jackdaw samples that were collected in the morning (09:29–10.41 h) versus in the afternoon (13:13–14:04), approximately 3.5 h apart (mean difference 3 h 31 min). One would expect that values of some of the blood analytes could differ between morning and afternoon, for example because nestlings that were blood sampled in the afternoon will have received more feeding visits by their parents and therefore had catabolized more food or due to diurnal rhythms in physiological parameters (Al-Haidary et al., 2016; Nichelmann et al., 1999).

### Effects of (short-term) handling stress

It is well known that blood analytes, e.g. glucose and protein levels, are affected by stress (Awerman and Romero, 2010; Viblanc et al., 2018) and this can obscure/mask the relationships between measured physiological parameters and ecological/behavioural traits. It is therefore important to quantify whether and to what extent Vetscan measurements of blood analytes are affected by short-time handling stress, which is often an issue when sampling wild animals under field conditions. We therefore collected three repeated samples from individual jackdaw nestlings, one within 3 min of taking them out of the nest box, one after the nestling had spent 16 min in darkness in a bird bag, and one after spending 30 min in a bird bag (Walker et al., 2005; Wingfield et al., 1997) (see also under sampling procedure above).

### Individual consistency in blood parameters over longer time periods

We used blood samples from zebra finches that we collected within a period of approximately 6 weeks (39–41 days) and within a period of approximately 1 year (366–371 days) to assess individual consistency of the different blood analytes over medium to long periods of time.

### Measurement errors

In total, we used 254 VetScan rotors in this study (62 on jackdaw samples, 192 on zebra finch samples). However, measurement of 19 (7.5%, four with jackdaw blood and 15 with zebra finch blood) rotors was cancelled during analysis due to the internal quality control system of the VetScan, most likely because some minor blood coagulation had occurred. The quality control system (QC) also reported 307 instances (16.9%, six jackdaw samples and 301 zebra finch samples) when the value of an analyte was outside the detection range [this concerned only the analytes bile acid (BA), uric acid (UA), total protein (TP) or albumin (ALB)]. The remaining 2278 values for blood analytes were analysed and reported without errors. The VetScan VS2 also reports hemolysis, lipemia, and icterus values as internal quality control [categorical values: 0 (clear), 1+ (slight), 2+ (moderate) and 3+(gross)]. Hemolysis, lipemia and icterus may interfere with analyses of some of the blood parameters (https://www.zoetis.es/_locale-assets/spc/vetscan-vs2-analizador.pdf). In our dataset, we found no QC error reports for hemolysis and lipemia, neither for jackdaws nor for zebra finches. The limited datasets suggest a significant increasing in hemolysis values for the long-term storage samples (hemolysis increased in seven out of eight samples, paired *t*-test, t=−4.9651, d.f.=7, *P*-value=0.002), although values were still within the detection range and approved by the QC. Jackdaw samples had icterus values that were always 0 or 1+ and, hence, did also not elicit an icterus warning. However, for the zebra finches the icterus values varied between 0 and 2+. For the UA analyses of zebra finch blood, 39.5% of the samples elicited a warning. Nevertheless, all UA values were within the VetScan dynamic ranges, and there were no significant differences in UA blood concentration between the different icterus categories (*F*_2, 86.52_=0.57, *P*=0.565) suggesting no strong interference of icterus with the measurements. To ensure that the high icterus did not interfere with the measurements in zebra finches, we conducted the statistical analyses (see below) with and without the individuals with high icterus vales and found no qualitative differences between the results. We therefore present the full dataset in the current study. For bile acid (BA), the Vetscan was not able to provide any qualified result for neither the jackdaws nor the zebra finches, and BA was therefore excluded from all analyses.

### Statistical analyses

Statistical analyses were performed using R 4.0.5 (R Core Team 2021). To determine if the repeatability of blood analytes differed between rotors (reproducibility), we calculated the repeatability of measurements based on the same blood sample but using different rotors (measurements taken immediately after each other) with the *rptR* package (version 0.9.22; Stoffel et al., 2017). Similarly, we calculated repeatability of VetScan measures from the same blood sample that were stored over a period of 12 h. To assess the effects of sampling time of the day, short-term (2 h) storage time and handling stress, we run general linear models or mixed models using the *lme4* package (version 1.1-28; Bates et al., 2015). If measurement values for a given blood analyte were significantly affected by storage effects or time of sampling in the previous models (see above), we included ‘time between sampling and analysis’ or ‘time of day’ (morning or afternoon) as additional predictor variables in the repeated measures model used to estimate the effect of handling stress. The Satterthwaite approximation was used to calculate the denominator degrees of freedom in mixed models. Means between different time points were compared with Tukey post-hoc tests. If residuals of the models were not normally distributed, we used non-parametric tests (Spearman rank correlation, Kruskal–Wallis and Wilcoxon signed ranks test) to assess differences between analyses or sampling points. The significance level was set at *P*<0.05. All tests were two-tailed.

### Ethical permits

Sampling of zebra finches and jackdaws was performed under permit from the Malmö/Lund ethical committee (permit no. 16-951/2018 and no. 18-0619/2019).

## References

[BIO060009C1] Aastrup, C. and Hegemann, A. (2021). Jackdaw nestlings rapidly increase innate immune function during the nestling phase but no evidence for a trade-off with growth. *Dev. Comp. Immunol.* 117, 103967. 10.1016/j.dci.2020.103967 33316356

[BIO060009C2] Aastrup, C., Nilsson, J. Å., Hasselquist, D. and Hegemann, A. (2023). Size and immune function as predictors of predation risk in nestling and newly fledged jackdaws. *Anim. Behav.* 198, 73-84. 10.1016/j.anbehav.2023.01.012

[BIO060009C3] Adamo, S. A. (2004). How should behavioural ecologists interpret measurements of immunity? *Anim. Behav.* 68, 1443-1449. 10.1016/j.anbehav.2004.05.005

[BIO060009C4] Adams, D., Gruber, E., Sather, H., Correa, M. and Crespo, R. (2022). Evaluation of growing turkey blood biochemistry panel measured using the VetScan VS2. *Poultry* 1, 138-146. 10.3390/poultry1020012

[BIO060009C5] Al-Haidary, A. A., Abdoun, K. A., Samara, E. M., Okab, A. B., Sani, M. and Refinetti, R. (2016). Daily rhythms of physiological parameters in the dromedary camel under natural and laboratory conditions. *Res. Vet. Sci.* 107, 273-277. 10.1016/j.rvsc.2016.07.006 27474007

[BIO060009C6] Ammersbach, M., Beaufrère, H., Gionet Rollick, A. and Tully, T. (2015). Laboratory blood analysis in Strigiformes-Part I: Hematologic reference intervals and agreement between manual blood cell counting techniques. *Vet. Clin. Pathol.* 44, 94-108. 10.1111/vcp.12229 25627556

[BIO060009C7] Andreasson, F., Nilsson, J. Å. and Nord, A. (2020). Avian reproduction in a warming world. *Front. Ecol. Evol.* 8, 576331. 10.3389/fevo.2020.576331

[BIO060009C8] Atkins, A., Jacobson, E., Hernandez, J., Bolten, A. B. and Lu, X. (2010). Use of a portable point-of-care (Vetscan Vs2) biochemical analyzer for measuring plasma biochemical levels in free-living loggerhead sea turtles (Caretta caretta). *J. Zoo Wildl. Med.* 41, 585-593. 10.1638/2009-0023.1 21370637

[BIO060009C9] Awerman, J. L. and Romero, L. M. (2010). Chronic psychological stress alters body weight and blood chemistry in European starlings (Sturnus vulgaris). *Comp. Biochem. Physiol. A Mol. Integr. Physiol.* 156, 136-142. 10.1016/j.cbpa.2010.01.010 20096363

[BIO060009C10] Azman, W. N. W., Omar, J., Koon, T. S. and Ismail, T. S. T. (2019). Hemolyzed specimens: Major challenge for identifying and rejecting specimens in clinical laboratories. *Oman Med. J.* 34, 94-98. 10.5001/omj.2019.19 30918601 PMC6425048

[BIO060009C11] Basile, A. J., Jasbi, P., Clark, W., Shi, X., Gu, H., Deviche, P. and Sweazea, K. L. (2020). A four-week white bread diet does not alter plasma glucose concentrations, metabolic or vascular physiology in mourning doves, Zenaida macroura. *Comp. Biochem. Physiol. A Mol. Integr. Physiol.* 247, 110718. 10.1016/j.cbpa.2020.110718 32376459

[BIO060009C12] Basile, A. J., Renner, M. W., Kayata, L., Deviche, P. and Sweazea, K. L. (2021). A four-week urban diet impairs vasodilation but not nutritional physiology in wild-caught mourning doves (Zenaida macroura). *Physiol. Biochem. Zool.* 94, 241-252. 10.1086/714831 34032554

[BIO060009C13] Bates, D., Mächler, M., Bolker, B. M. and Walker, S. C. (2015). Fitting linear mixed-effects models using lme4. *J. Stat. Softw.* 67, 1-48. 10.18637/jss.v067.i01

[BIO060009C14] Buehler, D. M., Piersma, T. and Irene Tieleman, B. (2008). Captive and free-living red knots Calidris canutus exhibit differences in non-induced immunity that suggest different immune strategies in different environments. *J. Avian Biol.* 39, 560-566. 10.1111/j.0908-8857.2008.04408.x

[BIO060009C15] Chaney, R. C., Blemings, K. P., Bonner, J. and Klandorf, H. (2003). Pentosidine as a measure of chronological age in wild birds. *Auk* 120, 394-399. 10.1093/auk/120.2.394

[BIO060009C16] Christopher, M. M. and O'neill, S. (2000). Effect of specimen collection and storage on blood glucose and lactate concentrations in healthy, hyperthyroid and diabetic cats. *Vet. Clin. Pathol.* 29, 22-28. 10.1111/j.1939-165X.2000.tb00386.x 12070820

[BIO060009C17] Cohen, A., Klasing, K. and Ricklefs, R. (2007). Measuring circulating antioxidants in wild birds. *Comp. Biochem. Physiol. B Biochem. Mol. Biol.* 147, 110-121. 10.1016/j.cbpb.2006.12.015 17303461

[BIO060009C18] Costantini, D. (2014). *Oxidative Stress and Hormesis in Evolutionary Ecology and Physiology: A Marriage Between Mechanistic and Evolutionary Approaches*. Springer Nature

[BIO060009C19] Criscuolo, F., Bize, P., Nasir, L., Metcalfe, N. B., Foote, C. G., Griffiths, K., Gault, E. A. and Monaghan, P. (2009). Real-time quantitative PCR assay for measurement of avian telomeres. *J. Avian Biol.* 40, 342-347. 10.1111/j.1600-048X.2008.04623.x

[BIO060009C20] Davies, S., Rodriguez, N. S., Sweazea, K. L. and Deviche, P. (2013). The effect of acute stress and long-term corticosteroid administration on plasma metabolites in an urban and desert songbird. *Physiol. Biochem. Zool.* 86, 47-60. 10.1086/667990 23303320

[BIO060009C21] Demas, G. E., Zysling, D. A., Beechler, B. R., Muehlenbein, M. P. and French, S. S. (2011). Beyond phytohaemagglutinin: Assessing vertebrate immune function across ecological contexts. *J. Anim. Ecol.* 80, 710-730. 10.1111/j.1365-2656.2011.01813.x 21401591

[BIO060009C22] Deviche, P., Beouche-Helias, B., Davies, S., Gao, S., Lane, S. and Valle, S. (2014). Regulation of plasma testosterone, corticosterone, and metabolites in response to stress, reproductive stage, and social challenges in a desert male songbird. *Gen. Comp. Endocrinol.* 203, 120-131. 10.1016/j.ygcen.2014.01.010 24518569

[BIO060009C23] Deviche, P., Valle, S., Gao, S., Davies, S., Bittner, S. and Carpentier, E. (2016). The seasonal glucocorticoid response of male Rufous-winged Sparrows to acute stress correlates with changes in plasma uric acid, but neither glucose nor testosterone. *Gen. Comp. Endocrinol.* 235, 78-88. 10.1016/j.ygcen.2016.06.011 27292791

[BIO060009C24] Dohm, M. R. (2002). Repeatability estimates do not always set an upper limit to heritability. *Funct. Ecol.* 16, 273-280. 10.1046/j.1365-2435.2002.00621.x

[BIO060009C25] Enders, L. S., Bickel, R. D., Brisson, J. A., Heng-Moss, T. M., Siegfried, B. D., Zera, A. J. and Miller, N. J. (2015). Abiotic and biotic stressors causing equivalent mortality induce highly variable transcriptional responses in the soybean aphid. *G3 (Bethesda)* 5, 261-270. 10.1534/g3.114.015149 PMC4321034 25538100

[BIO060009C26] Evans, S. R. and Gustafsson, L. (2017). Climate change upends selection on ornamentation in a wild bird. *Nat. Ecol. Evol.* 1, 39. 10.1038/s41559-016-0039 28812603

[BIO060009C27] Falconer, D. S. and Mackay, T. F. C. (1996). *Introduction To Quantitative Genetics 4th Edition : Falconer, Douglas S.: Free Download, Borrow, and Streaming : Internet Archive*, 4th edn. Essex, UK: Longman.

[BIO060009C28] Flatt, T. and Heyland, A. (2011). *Mechanisms of Life History Evolution: The Genetics and Physiology of Life History Traits and Trade-Offs*. Oxford University Press

[BIO060009C29] Fletcher, K., Xiong, Y., Fletcher, E. and Gustafsson, L. (2018). Glucocorticoid response to both predictable and unpredictable challenges detected as corticosterone metabolites in collared flycatcher droppings. *PLoS One* 13, e0209289. 30571789 10.1371/journal.pone.0209289 PMC6301662

[BIO060009C30] Greenacre, C. B., Flatland, B., Souza, M. J. and Fry, M. M. (2008). Comparison of avian biochemical test results with abaxis VetScan and Hitachi 911 analyzers. *J. Avian Med. Surg.* 22, 291-299. 10.1647/2007-034.1 19216256

[BIO060009C31] Gutiérrez, J. S., Sabat, P., Castañeda, L. E., Contreras, C., Navarrete, L., Peña-Villalobos, I. and Navedo, J. G. (2019). Oxidative status and metabolic profile in a long-lived bird preparing for extreme endurance migration. *Sci. Rep.* 9, 1-11. 10.1038/s41598-018-37186-2 31772390 PMC6879648

[BIO060009C33] Hasselquist, D., Wasson, M. F. and Winkler, D. W. (2001). Humoral immunocompetence correlates with date of egg-laying and reflects work load in female tree swallows. *Behav. Ecol.* 12, 93-97. 10.1093/oxfordjournals.beheco.a000384

[BIO060009C34] Hasselquist, D., Lindström, Å., Jenni-Eiermann, S., Koolhaas, A. and Piersma, T. (2007). Long flights do not influence immune responses of a long-distance migrant bird: a wind-tunnel experiment. *J. Exp. Biol.* 210, 1123-1131. 10.1242/jeb.02712 17371911

[BIO060009C35] Hegemann, A., Fudickar, A. M. and Nilsson, J. Å. (2019). A physiological perspective on the ecology and evolution of partial migration. *J. Ornithol.* 160, 893-905. 10.1007/s10336-019-01648-9

[BIO060009C36] Heins, M., Heil, W. and Withold, W. (1995). Storage of Serum or Whole Blood Samples? Effects of Time and Temperature on 22 Serum Analytes. *Clin. Chem. Lab. Med.* 33, 231-238. 10.1515/cclm.1995.33.4.231 7626695

[BIO060009C37] Heireman, L., Van Geel, P., Musger, L., Heylen, E., Uyttenbroeck, W. and Mahieu, B. (2017). Causes, consequences and management of sample hemolysis in the clinical laboratory. *Clin. Biochem.* 50, 1317-1322. 10.1016/j.clinbiochem.2017.09.013 28947321

[BIO060009C38] Hoppes, S. M., Boyd, J. D. and Brightsmith, D. J. (2015). Impact of delayed analysis in avian blood biochemical values measured with the abaxis VetScan VS2. *J. Avian Med. Surg.* 29, 200-209. 10.1647/2014-033 26378666

[BIO060009C39] Huber, N., Fusani, L., Ferretti, A., Mahr, K. and Canoine, V. (2017). Measuring short-term stress in birds: Comparing different endpoints of the endocrine-immune interface. *Physiol. Behav.* 182, 46-53. 10.1016/j.physbeh.2017.09.017 28958953

[BIO060009C40] Kachhawa, K., Kachhawa, P., Varma, M., Behera, R., Agrawal, D. and Kumar, S. (2017). Study of the stability of various biochemical analytes in samples stored at different predefined storage conditions at an accredited laboratory of India. *J. Lab. Physicians* 9, 011-015. 10.4103/0974-2727.187928 PMC5015491 28042210

[BIO060009C42] Lee, J. E., Hong, M., Park, S. K., Yu, J. I., Wang, J. S., Shin, H., Kim, J. W., Han, B. G. and Shin, S. Y. (2016). Inorganic Phosphorus and Potassium Are Putative Indicators of Delayed Separation of Whole Blood. *Osong Public Heal. Res. Perspect.* 7, 90-95. 10.1016/j.phrp.2015.11.003 PMC4850501 27169006

[BIO060009C43] Lettoof, D. C., Aubret, F., Spilsbury, F., Bateman, P. W., Haberfield, J., Vos, J. and Gagnon, M. M. (2021). Plasma biochemistry profiles of wild western tiger snakes (Notechis scutatus occidentalis) before and after six months of captivity. *J. Wildl. Dis.* 57, 253-263. 10.7589/JWD-D-20-00115 33822160

[BIO060009C44] Li, M., Zhu, W., Wang, Y., Sun, Y., Li, J., Liu, X., Wu, Y., Gao, X. and Li, D. (2019). Effects of capture and captivity on plasma corticosterone and metabolite levels in breeding Eurasian Tree Sparrows. *Avian Res.* 10, 16. 10.1186/s40657-019-0155-8

[BIO060009C45] Männistö, T., Surcel, H. M., Bloigu, A., Ruokonen, A., Hartikainen, A. L., Järvelin, M. R., Pouta, A., Vääräsmäki, M. and Suvanto-Luukkonen, E. (2007). The effect of freezing, thawing, and short- and long-term storage on serum thyrotropin, thyroid hormones, and thyroid autoantibodies: Implications for analyzing samples stored in serum banks [7]. *Clin. Chem.* 53, 1986-1987. 10.1373/clinchem.2007.091371 17954505

[BIO060009C46] Matson, K. D., Ricklefs, R. E. and Klasing, K. C. (2005). A hemolysis–hemagglutination assay for characterizing constitutive innate humoral immunity in wild and domestic birds. *Dev. Comp. Immunol.* 29, 275-286. 10.1016/j.dci.2004.07.006 15572075

[BIO060009C47] Matson, K. D., Cohen, A. A., Klasing, K. C., Ricklefs, R. E. and Scheuerlein, A. (2006). No simple answers for ecological immunology: Relationships among immune indices at the individual level break down at the species level in waterfowl. *Proc. R. Soc. B Biol. Sci.* 273, 815-822. 10.1098/rspb.2005.3376 PMC1560229 16618674

[BIO060009C48] Mcwilliams, S., Carter, W., Cooper-Mullin, C., Demoranville, K., Frawley, A., Pierce, B. and Skrip, M. (2021). How birds during migration maintain (Oxidative) balance. *Front. Ecol. Evol.* 9, obab035. 10.3389/fevo.2021.742642

[BIO060009C49] Morales, A., Frei, B., Leung, C., Titman, R., Whelan, S., Benowitz-Fredericks, Z. M. and Elliott, K. H. (2020). Point-of-care blood analyzers measure the nutritional state of eighteen free-living bird species. *Comp. Biochem. Physiol. A Mol. Integr. Physiol.* 240, 110594. 10.1016/j.cbpa.2019.110594 31676409

[BIO060009C50] Nichelmann, M., Höchel, J. and Tzschentke, B. (1999). Biological rhythms in birds - Development, insights and perspectives. *Comp. Biochem. Physiol. A Mol. Integr. Physiol.* 124, 429-437. 10.1016/S1095-6433(99)00135-X 10682241

[BIO060009C51] Nordfjäll, K., Larefalk, Å., Lindgren, P., Holmberg, D. and Roos, G. (2005). Telomere length and heredity: Indications of paternal inheritance. *Proc. Natl. Acad. Sci. U. S. A.* 102, 16374-16378. 10.1073/pnas.0501724102 16258070 PMC1283414

[BIO060009C52] Oddoze, C., Lombard, E. and Portugal, H. (2012). Stability study of 81 analytes in human whole blood, in serum and in plasma. *Clin. Biochem.* 45, 464-469. 10.1016/j.clinbiochem.2012.01.012 22285385

[BIO060009C53] Peruffo L, Boyd J. D., Hoppes S., Brightsmith D. J. (2016). Blood Biochemical Values of Wild Scarlet Macaw (*Ara macao macao*) Nestlings and Adults. *J. Avian Med. Surg**.* 30, 227-236. 10.1647/2015-115 27736226

[BIO060009C54] Ruiz-Jimenez, F., Gruber, E., Correa, M. and Crespo, R. (2021). Comparison of portable and conventional laboratory analyzers for biochemical tests in chickens. *Poult. Sci.* 100, 746-754. 10.1016/j.psj.2020.11.060 33518128 PMC7858187

[BIO060009C55] Sautin, Y. Y. and Johnson, R. J. (2008). Uric acid: The oxidant-antioxidant paradox. *Nucleosides Nucleotides Nucleic Acids* 27, 608-619. 10.1080/15257770802138558 18600514 PMC2895915

[BIO060009C56] Schoepf, I., Pillay, N. and Schradin, C. (2017). Trade-offs between reproduction and health in free-ranging African striped mice. *J. Comp. Physiol. B Biochem. Syst. Environ. Physiol.* 187, 625-637. 10.1007/s00360-016-1054-5 28161790

[BIO060009C57] Scope, A., Filip, T., Gabler, C. and Resch, F. (2002). The influence of stress from transport and handling on hematologic and clinical chemistry blood parameters of racing pigeons (Columba livia domestica). *Avian Dis.* 46, 224-229. 10.1637/0005-2086(2002)046[0224:TIOSFT]2.0.CO;2 11922340

[BIO060009C58] Sørensen, S. L., Park, Y., Gong, Y., Vasanth, G. K., Dahle, D., Korsnes, K., Phuong, T. H., Kiron, V., Øyen, S., Pittman, K. et al. (2021). Nutrient digestibility, growth, mucosal barrier status, and activity of leucocytes from head kidney of atlantic salmon fed marine- or plant-derived protein and lipid sources. *Front. Immunol.* 11, 3939. 10.3389/fimmu.2020.623726 PMC7934624 33679713

[BIO060009C59] Stoffel, M. A., Nakagawa, S. and Schielzeth, H. (2017). rptR: repeatability estimation and variance decomposition by generalized linear mixed-effects models. *Methods Ecol. Evol.* 8, 1639-1644. 10.1111/2041-210X.12797

[BIO060009C60] Strazzullo, P. and Puig, J. G. (2007). Uric acid and cardiovascular risk: Is the devil always so bad? *Nutr. Metab. Cardiovasc. Dis.* 17, 409-414. 10.1016/j.numecd.2007.02.011 17643880

[BIO060009C61] Turchiano, M., Nguyen, C., Fierman, A., Lifshitz, M. and Convit, A. (2013). Impact of blood sample collection and processing methods on glucose levels in community outreach studies. *J. Environ. Public Health* 2013, 1-4. 10.1155/2013/256151 PMC3556871 23365588

[BIO060009C62] Vágási, C. I., Vincze, O., Pătraș, L., Osváth, G., Pénzes, J., Haussmann, M. F., Barta, Z. and Pap, P. L. (2019). Longevity and life history coevolve with oxidative stress in birds. *Funct. Ecol.* 33, 152-161. 10.1111/1365-2435.13228 34290466 PMC8291348

[BIO060009C63] Viblanc, V. A., Schull, Q., Cornioley, T., Stier, A., Ménard, J. J., Groscolas, R. and Robin, J. P. (2018). An integrative appraisal of the hormonal and metabolic changes induced by acute stress using king penguins as a model. *Gen. Comp. Endocrinol.* 269, 1-10. 10.1016/j.ygcen.2017.08.024 28843614

[BIO060009C64] Walker, B. G., Boersma, P. D. and Wingfield, J. C. (2005). Field endocrinology and conservation biology. *Integr. Comp. Biol.* 45, 12-18. 10.1093/icb/45.1.12 21676739

[BIO060009C65] Wingfield, J. C. and Farner, D. S. (1975). The determination of five steroids in avian plasma by radioimmunoassay and competitive protein-binding. *Steroids* 26, 311-327. 10.1016/0039-128X(75)90077-X 1198621

[BIO060009C66] Wingfield, J. C., Hunt, K., Breuner, C., Dunlap, K., Fowler, G. S., Freed, L., Lepson, J., Clemmons, J. R. and Buchholz, R. (1997). Behavioral approaches to conservation in the wild. *Behav. Approaches to Conserv. Wild* 95-131.

[BIO060009C67] Zylberberg, M. (2015). Common measures of immune function vary with time of day and sampling protocol in five passerine species. *J. Exp. Biol.* 218, 757-766. 10.1242/jeb.111716 25617452

